# miRNA-200c-3p is crucial in acute respiratory distress syndrome

**DOI:** 10.1038/celldisc.2017.21

**Published:** 2017-06-27

**Authors:** Qiang Liu, Jianchao Du, Xuezhong Yu, Jun Xu, Fengming Huang, Xiaoyun Li, Cong Zhang, Xiao Li, Jiahui Chang, Daozhen Shang, Yan Zhao, Mingyao Tian, Huijun Lu, Jiantao Xu, Chang Li, Huadong Zhu, Ningyi Jin, Chengyu Jiang

**Affiliations:** 1 Department of Biochemistry, State Key Laboratory of Medical Molecular Biology, Institute of Basic Medical Sciences, Chinese Academy of Medical Sciences, Peking Union Medical College, Beijing, China; 2 Department of Emergency, Peking Union Medical College Hospital, Chinese Academy of Medical Sciences, Beijing, China; 3 School of Life Science, University of Science and Technology of China, Hefei, China; 4 Genetic Engineering Laboratory, Institute of Military Veterinary, Academy of Military Medical Sciences, Changchun, China; 5 State Key Laboratory of Biotherapy/Collaborative Innovation Center for Biotherapy, West China Hospital, Sichuan University, Chengdu, China

**Keywords:** influenza, poly (I:C), LPS, LTA, miRNA, pneumonia, angiotensin

## Abstract

Influenza infection and pneumonia are known to cause much of their mortality by inducing acute respiratory distress syndrome (ARDS), which is the most severe form of acute lung injury (ALI). Angiotensin-converting enzyme 2 (ACE2), which is a negative regulator of angiotensin II in the renin–angiotensin system, has been reported to have a crucial role in ALI. Downregulation of ACE2 is always associated with the ALI or ARDS induced by avian influenza virus, severe acute respiratory syndrome-coronavirus, respiratory syncytial virus and sepsis. However, the molecular mechanism of the decreased expression of ACE2 in ALI is unclear. Here we show that avian influenza virus H5N1 induced the upregulation of miR-200c-3p, which was then demonstrated to target the 3′-untranslated region of ACE2. Then, we found that nonstructural protein 1 and viral RNA of H5N1 contributed to the induction of miR-200c-3p during viral infection. Additionally, the synthetic analog of viral double-stranded RNA (poly (I:C)), bacterial lipopolysaccharide and lipoteichoic acid can all markedly increase the expression of miR-200c-3p in a nuclear factor**-**κB-dependent manner. Furthermore, markedly elevated plasma levels of miR-200c-3p were observed in severe pneumonia patients. The inhibition of miR-200c-3p ameliorated the ALI induced by H5N1 virus infection *in vivo*, indicating a potential therapeutic target. Therefore, we identify a shared mechanism of viral and bacterial lung infection-induced ALI/ARDS via nuclear factor-κB-dependent upregulation of miR-200c-3p to reduce ACE2 levels, which leads increased angiotensin II levels and subsequently causes lung injury.

## Introduction

Acute respiratory distress syndrome (ARDS), the most severe form of acute lung injury (ALI), is the main predisposing factor in highly pathogenic avian influenza virus-induced death cases [1, 2]. The ALI caused by influenza infection can facilitate bacterial superinfection, which is a major factor that promotes mortality and disease severity [3]. From 2003 to 2016, highly pathogenic avian H5N1 influenza virus has caused 856 human infection cases worldwide with a high mortality rate of 52.8% (http://101.96.8.164/www.who.int/entity/influenza/human_animal_interface/2017_01_16_tableH5N1.pdf). Currently, China is undergoing its fifth epidemic of human infections of avian H7N9 influenza virus. Until 16 January 2017, 918 H7N9-infected human cases with at least 359 deaths have been reported to World Health Organization (http://101.96.8.165/www.who.int/influenza/human_animal_interface/Influenza_Summary_IRA_HA_interface_01_16_2017_FINAL.pdf?ua=1). With high mutation and reassortment rates, influenza viruses have evolved with a rapid resistance to the current vaccines and anti-viral drugs, making an avian influenza pandemic an urgent unresolved threat to human health [4–6].

Angiotensin-converting enzyme 2 (ACE2) was firstly identified from human cardiac left ventricle complementary DNA (cDNA) library and lymphoma cDNA library by two separate groups [7, 8]. ACE2 inactivates angiotensin II (Ang II) by cleaving it to produce Ang 1–7 [9]. Ang II binds to the Ang II type 1 and type 2 receptors with strong affinity, mediating regulation of blood pressure, body fluid balance, inflammation, cell proliferation, hypertrophy and fibrosis [10–12]. Reduced expression levels of ACE2 have been reported in hypertension and chronic kidney disease [13, 14]. In addition, ACE2 was identified as a key regulator of dietary amino-acid homeostasis and gut microbial ecology [15]. We previously demonstrated that ACE2 counteracts the development of severe ALI or ARDS induced by avian influenza virus, severe acute respiratory syndrome-coronavirus spikes, sepsis and acid aspiration in mice [16–18]. Administration of recombinant ACE2 or the Ang II receptor blocker losartan can ameliorate H5N1 virus infection-induced ALI [16, 19]. Moreover, ACE2 was also reported to have a protective role in respiratory syncytial virus-induced lung injury [20]. Notably, plasma Ang II levels can indicate disease severity in H7N9 infections [21]. Downregulation of ACE2 appears to be a shared phenomenon in ALI or ARDS induced by viral or bacterial infection [18, 22–25].

MicroRNAs (miRNAs) are small non-coding RNAs that function primarily in regulating gene expression by binding to the 3′-untranslated region (3′-UTR) of the targeted mRNAs [26]. Wide-spectrum diseases, such as pulmonary diseases, diabetes and cardiac disorders, can be attributed to miRNA dysfunction [27]. In recent years, multiple miRNA-based drugs have been explored and have entered clinical testing [27, 28]. miR-200 family consists of two clusters: the miR-200a/b/429 cluster containing miR-200a-3p, miR-200a-5p, miR-200b-3p, miR-200b-5p and miR-429 on chromosome 1p36.33, and the miR-200c/141 cluster containing miR-200c-3p, miR-200c-5p, miR-141-3p and miR-141-5p on chromosome 12p13.31. Studies on this miRNA family revealed that it has versatile roles in cancer progression, drug resistance and oxidative stress [29, 30].

In our study, we proposed to explore the mechanism behind the downregulation of ACE2 presenting in ALI or ARDS induced by avian influenza virus or other pathogens. We demonstrated that miR-200c-3p was markedly upregulated by the infection of H5N1 virus and the treatment of synthetic double-stranded RNA poly (I:C), bacterial lipopolysaccharide (LPS) and lipoteichoic acid (LTA) in a nuclear factor-κB (NF-κB)-dependent manner. miR-200c-3p directly targets the 3′-UTR of ACE2 and downregulates ACE2 protein expression. Moreover, elevated levels of miR-200c-3p were found in plasma samples of severe pneumonia patients. Our *in vivo* results demonstrated that miR-200c-3p may be a therapeutic target for ALI.

## Results

### H5N1 infection induces the upregulation of miR-200c-3p in A549 cells

**Figure 1:**
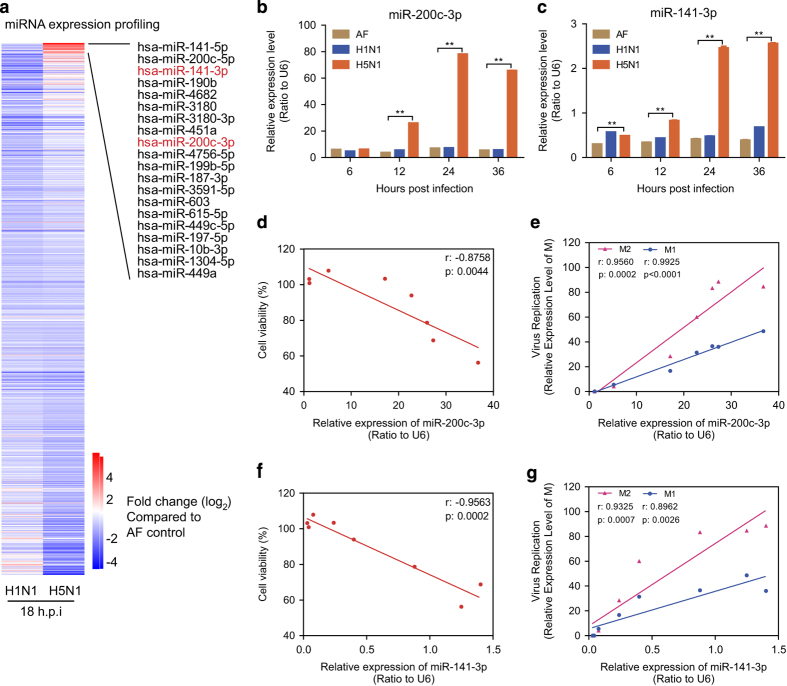
Aberrant expression of miR-200c-3p and miR-141-3p in H5N1-infected A549 cells. (**a**) Heat map of fold changes (log 2) of miRNAs’ expression in A549 cells after infection with either H1N1 or H5N1 (MOI=4) influenza virus for 18 h. The fold changes were compared with miRNAs’ expression in cells mock infected with AF for 18 h. The upregulated miRNAs in H5N1-infected A549 cells are listed. (**b** and **c**) qRT-PCR analysis of the expression of miR-200c-3p and miR-141-3p in A549 cells at the indicated hours after challenge with H1N1 virus, H5N1 virus (MOI=4) or AF control. Graph shows the mean±s.e.m. **P<0.01. (**d** and **e**) Correlation between the expression of miR-200c-3p and cell viability or virus replication in H5N1-infected A549 cells. Pearson's correlation analysis was used to analyze the correlation. Pearson's correlation coefficients (*r*) and *P*-values are provided in each graph. Viral replication was indicated with the relative expression levels of M1 and M2. (**f** and **g**) Correlation between the expression of miR-141-3p and cell viability or virus replication in H5N1-infected A549 cells. Pearson's correlation analysis was used to analyze the correlation. Pearson's correlation coefficients (*r*) and *P*-values are provided in each graph.

To identify miRNAs potentially regulating ACE2 protein expression, the miRNA expression profile in A549 cells (human lung adenocarcinoma epithelial cell line) challenged with H1N1 or H5N1 influenza virus was compared with that in A549 cells mock infected with allantoic fluid (AF). With 978 human miRNAs at a meaningful level (read count>0), we listed the top 20 highly expressed miRNAs in H5N1-infected cells compared with H1N1-infected cells (Figure 1a). Among them, miR-200c-3p and miR-141-3p were predicted to target ACE2 using TargetScan (Supplementary Figure S1a). Quantitative real-time PCR (qRT-PCR) analysis confirmed the upregulation of miR-200c-3p and miR-141-3p in H5N1-infected A549 cells (Figure 1b and c). Moreover, we observed that the expression of miR-421, which was reported to target ACE2 [31], was not upregulated after H1N1 and H5N1 virus infection (Supplementary Figure S1b). The pattern of the upregulation of miR-200c-3p and miR-141-3p was also validated in HEK293T cells (human embryonic kidney cells) (Supplementary Figure S1c and d). Additionally, the ACE2 protein expression levels were downregulated in A549 cells (Supplementary Figure S1e) and HEK293T cells (Supplementary Figure S1f) after infection with H5N1 influenza virus, which were consistent with our previous observations in the H5N1-infected mice lung tissues [16].

Interestingly, the H5N1-induced expression of miR-200c-3p and miR-141-3p was highly correlated with both cell viability (Figure 1d and f) and viral replication (Figure 1e and g). However, the expression of neither miR-200c-5p nor miR-141-5p was correlated with cell viability or viral replication (Supplementary Figure S1g and h). Then, we tested the effect of the different multiplicity of infection (MOI) on the expression of miR-200c-3p and miR-141-3p. A549 cells were challenged with H5N1 virus at different viral titers (MOI=0.05, 0.5 and 5), and the expression of miR-200c-3p and miR-141-3p was detected. The larger the infective dose administered, the higher the expression of miR-200c-3p and miR-141-3p (Supplementary Figure S1i). Successful viral infection was confirmed by detecting the expression of matrix protein 1 (M1) (Supplementary Figure S1j). Collectively, miR-200c-3p and miR-141-3p may be capable of regulating the expression of ACE2 protein and have a critical role during H5N1 virus infection.

### miR-200c-3p downregulates ACE2 expression by directly targeting to the 3′-UTR of ACE2

**Figure 2:**
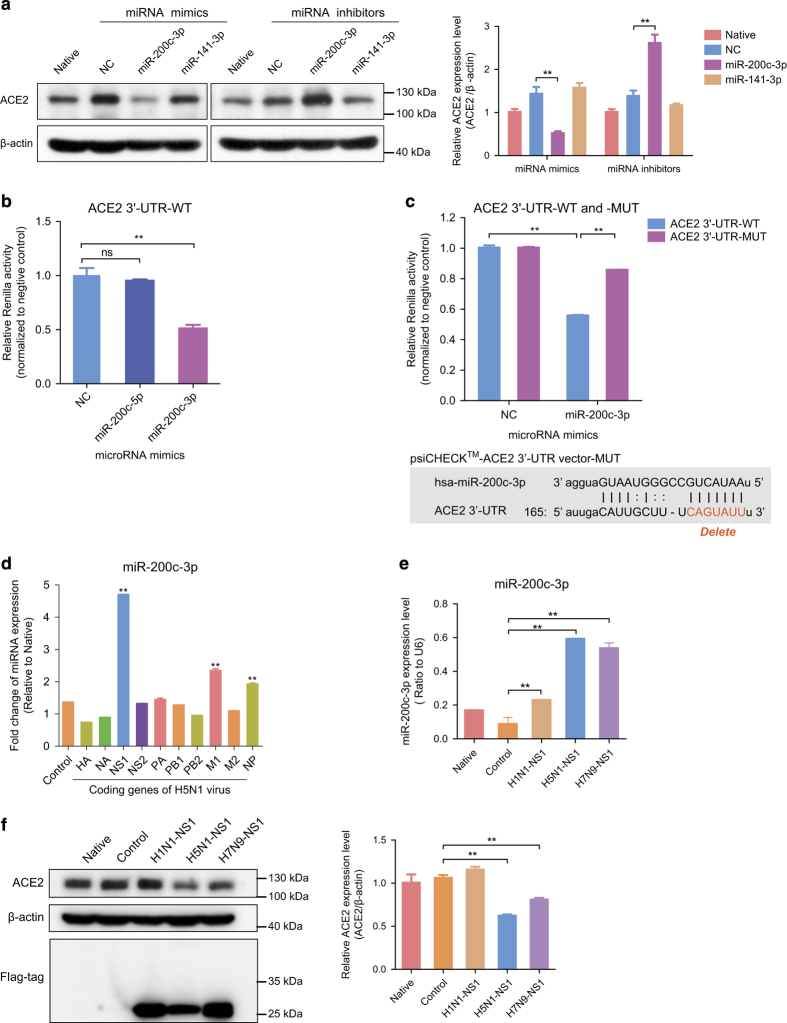
ACE2 is a target of miR-200c-3p that can be induced by NS1. (**a**) A549 cells were transfected with 50 nM mimics or inhibitors of miR-200c-3p or miR-141-3p for 36 h. ACE2 protein expression levels were analyzed by western blotting and were normalized to β-actin. Native, untreated A549 cells. (**b**) The wild-type 3′-UTR of the ACE2 transcript was cloned into the psiCHECK-2 vector (ACE2 3′-UTR-WT reporter vector). Luciferase activity in HEK293T cells transfected with mimics of miRNAs or the vector was detected using the Dual Luciferase Reporter Assay System. (**c**) An ACE2 3′-UTR-MUT reporter vector was constructed in which the miR-200c-3p binding site on the ACE2 3′-UTR was deleted. Luciferase activity in HEK293T cells transfected with miR-200c-3p mimics and the ACE2 3′-UTR-WT or -MUT reporter vector was detected using the Dual Luciferase Reporter Assay System. (**d**) Vectors containing H5N1 influenza viral protein coding genes were individually transfected into HEK293T cells for 48 h. The expression of miR-200c-3p was quantified by qRT-PCR. Native, untreated HEK293T cells. (**e** and **f**) Vectors coding for NS1 of H1N1, H5N1 and H7N9 viruses were transfected into HEK293T cells for 72 h. The expression of miR-200c-3p was detected by qRT-PCR. The expression of ACE2 and NS1 was detected by western blotting. In each graph, the data are presented as the mean±s.e.m. NS, not significant, **P<0.01.

In accordance with the prediction, transfection of miR-200c-3p mimics into A549 cells significantly decreased the abundance of ACE2 protein, whereas miR-200c-3p inhibitors increased it (Figure 2a). However, we did not observe any significant change of ACE2 expression in the group transfected with miR-141-3p mimics or inhibitors. Previously, miR-141-3p has been reported to regulate tumor growth factor-β2 by targeting its transcript during H5N1 infection [32]. To demonstrate the interaction between miR-200c-3p and ACE2 transcripts, we constructed a reporter vector in which the 3′-UTR of ACE2 was fused downstream of *Renilla* luciferase coding sequence (Supplementary Figure S2a). In HEK293T cells transfected with miR-200c-3p mimics rather than miR-200c-5p mimics, *Renilla* luciferase activity significantly decreased (Figure 2b). Successful transfection of miR-200c-3p mimics and miR-200c-5p mimics was confirmed by qRT-PCR (Supplementary Figure S2b). Additionally, the activity was inhibited by miR-200c-3p mimics in a concentration-dependent manner (Supplementary Figure S2c). When the predicted miR-200c-3p binding site on ACE2 3′-UTR was deleted, the inhibiting effects of miR-200c-3p mimics on the luciferase activity were diminished (Figure 2c). Moreover, mutation of the site on miR-200c-3p binding to ACE2 3′-UTR abolished the inhibiting effects of miR-200c-3p on *Renilla* luciferase activity (Supplementary Figure S2d). Taken together, these results indicate that miR-200c-3p, rather than miR-200c-5p and miR-141-3p, directly targets the 3′-UTR of ACE2 and downregulates the expression of ACE2 protein.

### NS1 of H5N1 and H7N9 viruses can induce the upregulation of miR-200c-3p

To elucidate how H5N1 influenza virus induces the upregulation of miR-200c-3p, vectors containing the H5N1 influenza viral protein coding genes, hemagglutinin, neuraminidase (NA), nonstructural protein 1 (NS1), nonstructural protein 2 (NS2), polymerase complex PA, polymerase complex PB1, polymerase complex PB2, M1, matrix protein 2 (M2) and nucleocapsid protein (NP) were each transfected into HEK293T cells for 48 h. In HEK293T cells transfected with NS1 expressing vector, the upregulation of miR-200c-3p and miR-141-3p was observed (Figure 2d and Supplementary Figure S3a). In addition, M1 and NP also induced the upregulation of miR-200c-3p. However, NS1 induced a much higher level of miR-200c-3p than M1 and NP. Therefore, in the subsequent experiments, we focused on the function of NS1. Compared with the NS1 of H1N1 virus, the NS1 of H5N1 and H7N9 viruses induced much higher expression of miR-200c-3p and miR-141-3p (Figure 2e and Supplementary Figure S3b). However, NS1 of neither H5N1 nor H7N9 caused any significant change of the expression of miR-421 (Supplementary Figure S3c). Transfection of H5N1-NS1 and H7N9-NS1 expression vectors into HEK293T cells resulted in the downregulation of ACE2 protein levels (Figure 2f). Our results suggest that NS1 of pathogenic avian influenza virus has an important role in the induction of miR-200c-3p and miR-141-3p.

### Induction of miR-200c-3p in cells treated with poly (I:C), and bacterial components LPS and LTA

**Figure 3:**
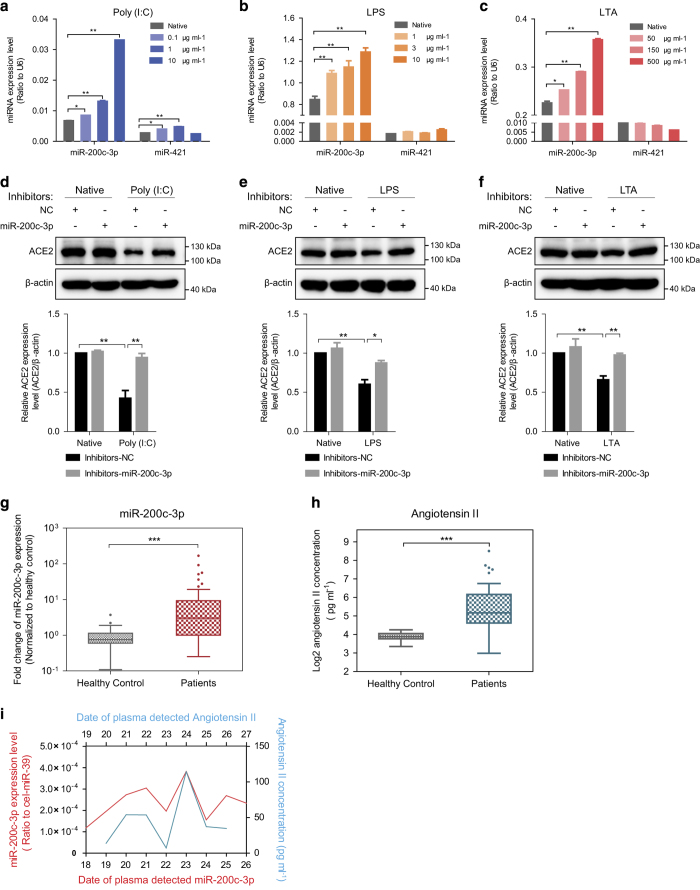
Elevated levels of miR-200c-3p in cells treated with poly (I:C), LPS and LTA and in the plasma of severe pneumonia patients. (**a**) qRT-PCR analysis of the expression of miR-200c-3p and miR-421 in A549 cells transfected with poly (I:C) for 6 h at the indicated concentration. (**b**) qRT-PCR analysis of the expression of miR-200c-3p and miR-421 in A549 cells treated with LPS for 24 h at the indicated concentration. (**c**) qRT-PCR analysis of the expression of miR-200c-3p and miR-421 in A549 cells treated with LTA for 24 h at the indicated concentration. (**d**) After transfection with inhibitors of NC or miR-200c-3p for 6 h, A549 cells were transfected with poly (I:C) (10 μg ml^−1^). The expression of ACE2 protein in the cells was detected. Native, A549 cells were treated with the solvent used. (**e**) After transfection with inhibitors of NC or miR-200c-3p for 6 h, A549 cells were challenged with LPS (10 μg ml^−1^). The expression of ACE2 protein in the cells was detected. Native, A549 cells were treated with the solvent used. (**f**) After transfected with inhibitors of NC or miR-200c-3p for 6 h, A549 cells were challenged with LTA at (500 μg ml^−1^). The expression of ACE2 protein in the cells was detected. β-Actin served as an internal control. Native, A549 cells were treated with the solvent used. (**g** and **h**) qRT-PCR analysis of miR-200c-3p (**g**) and enzyme-linked immunosorbent assay analysis of Ang II (**h**) in the plasma of healthy controls and severe pneumonia patients. The number of the study participants for each group was as follows: healthy control group (*n*=21), severe pneumonia patients (*n*=56). (**i**) Kinetics of miR-200c-3p (ratio to *cel-miR-39* which was used as a spike-in control) and Ang II plasma levels of one severe pneumonia patient (patient 48th). The data are shown as the mean±s.e.m. **P<0.01 and ***P<0.001.

To examine whether the induction of miR-200c-3p to downregulate ACE2 is a specific mechanism of pathogenic avian influenza viruses, we challenged A549 cells with poly (I:C). miR-200c-3p was upregulated in a concentration-dependent manner in poly (I:C)-transfected A549 cells (Figure 3a). In addition, transfection of poly (I:C) also resulted in the downregulation of ACE2 protein expression, which was rescued by the transfection of inhibitors of miR-200c-3p (Figure 3d), indicating that downregulating ACE2 protein expression by miR-200c-3p may be a shared mechanism for RNA viruses. Induction of interleukin-6, interleukin-29 and interferon-β by poly (I:C) was confirmed by qRT-PCR analysis (Supplementary Figure S4a). Previous studies have reported that LPS, which is the major constituent of the outer membrane of Gram-negative bacteria, can induce the downregulation of ACE2 and the upregulation of Ang II [33, 34]. However, the mechanisms are still unclear. By exposing A549 and THP1 cells (human acute monocytic leukemia cell line) to LPS, we found that LPS can induce the upregulation of miR-200c-3p (Figure 3b and Supplementary Figure S5a). LPS treatment also induced the downregulation of ACE2 protein expression (Figure 3e and Supplementary Figure S5c). Compared with inhibitors of negative control (NC), transfection of miR-200c-3p inhibitors significantly inhibited the downregulation of ACE2 induced by LPS (Figure 3e). Interestingly, LTA, which is the major component of the cell wall of Gram-positive bacteria, can also induce the upregulation of miR-200c-3p (Figure 3c and Supplementary Figure S5b) and the downregulation of ACE2 (Figure 3f and Supplementary Figure S5d) in A549 and THP1 cells. Consistently, the downregulation of ACE2 protein was markedly inhibited by the transfection of miR-200c-3p inhibitors (Figure 3f). In poly (I:C)-treated cells, another miRNA previously reported targeting to ACE2 gene, miR-421, was measured slightly upregulated, whereas in LPS- and LTA-treated A549 cells, no significant changes of miR-421 were observed (Figure 3a–c). Induction of tumor necrosis factor-α, interleukin-6 and interleukin-1β by LPS and LTA in A549 and THP1 cells were confirmed by qRT-PCR analysis (Supplementary Figure S4b and c and Supplementary Figure S5e and f). Collectively, our results suggest that miR-200c-3p is a crucial regulator of ACE2 protein expression in the above conditions and may be shared in certain types of viral and bacterial infections.

### Elevated plasma levels of miR-200c-3p in severe pneumonia patients

Viruses and bacteria are usually the precipitating factors of severe pneumonia, which may induce severe respiratory failure by triggering ARDS [35, 36], a clinical syndrome characterized by pulmonary edema, hypoxemia and the accumulation of inflammatory cells [1]. ARDS has a high mortality rate of 30–50%, thus demanding accurate prediction models [37, 38]. Because H5N1 virus, poly (I:C), LPS and LTA can all induce the expression of miR-200c-3p to downregulate ACE2 protein expression, we speculate that the expression of miR-200c-3p may be aberrant in severe pneumonia infection. To investigate whether miR-200c-3p is involved in severe pneumonia infection, we collected plasma from 56 severe pneumonia patients and 21 healthy volunteers and then detected the expression of miR-200c-3p in the plasma. The characteristics of the study subjects are shown in Supplementary Table S1. Of the 56 patients, 53 (94.64%) fulfilled ARDS criteria [39]. In accordance with our speculation, the plasma levels of miR-200c-3p were significantly elevated in severe pneumonia patients (Figure 3g). Downregulation of ACE2 may result in the elevated plasma level of Ang II, which is a major substrate of ACE2. Additionally, Ang II is claimed considerably increased in ARDS patients [40]. Consistent with this report, the plasma levels of Ang II were also markedly upregulated in severe pneumonia patients (Figure 3h). Notably, the plasma level of Ang II changed over miR-200c-3p in severe pneumonia patient (patient 48th) whose plasma samples were consecutively collected (Figure 3i), suggesting that miR-200c-3p may participate in the upregulation of plasma Ang II and then induces lung injury through Ang II type 1 receptor. Consequently, our results raise the possibility that miR-200c-3p can serve as a biomarker or therapeutic target for severe pneumonia.

### NF-κB signaling pathway mediates the upregulation of miR-200c-3p induced by H5N1, poly (I:C), LPS and LTA

**Figure 4:**
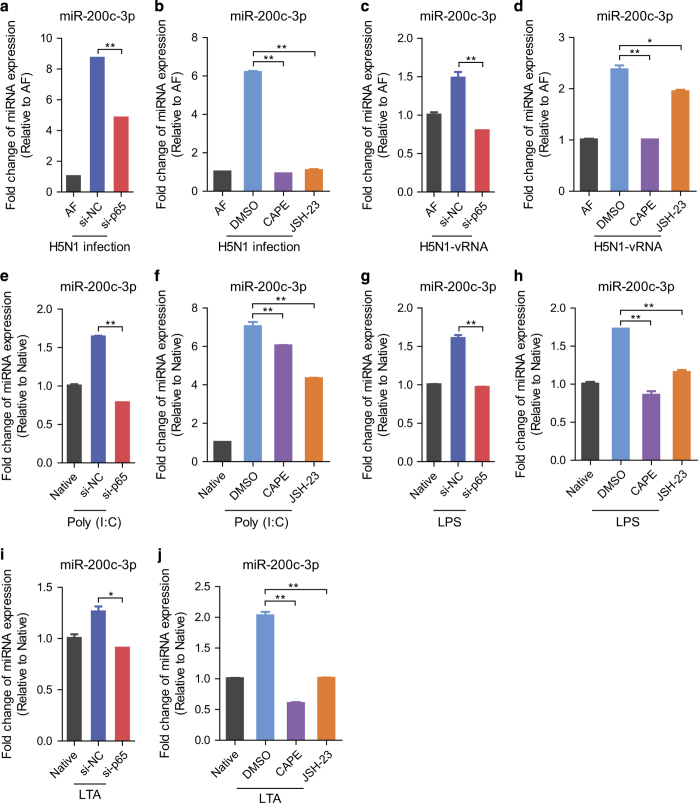
NF-κB signaling pathway mediates the upregulation of miR-200c-3p. (**a**) A549 cells were transfected with the corresponding siRNAs and then challenged with AF or H5N1 (MOI=4) for 48 h. The expression of miR-200c-3p was detected by qRT-PCR. (**b**) A549 cells were treated with CAPE or JSH-23 and then challenged with AF or H5N1 (MOI=4) for 48 h. The expression of miR-200c-3p was detected by qRT-PCR. (**c**) A549 cells were transfected with the corresponding siRNAs and then transfected with 1 μg ml^−1^ RNA extracted from AF- or H5N1-infected cells for 12 h. The expression of miR-200c-3p was detected by qRT-PCR. (**d**) A549 cells were treated with CAPE or JSH-23 and then transfected with 1 μg ml^−1^ RNA extracted from AF- or H5N1-infected cells for 12 h. The expression of miR-200c-3p was detected by qRT-PCR. (**e**) A549 cells were transfected with the corresponding siRNAs and then transfected with 1 μg ml^−1^ poly (I:C) for 12 h. The expression of miR-200c-3p was detected by qRT-PCR. Native, A549 cells were treated with the solvent used. (**f**) A549 cells were treated with CAPE or JSH-23 and then transfected with 1 μg ml^−1^ poly (I:C) for 12 h. The expression of miR-200c-3p was detected by qRT-PCR. Native, A549 cells were treated with the solvent used. (**g**) THP1 cells were transfected with the corresponding siRNAs and then treated with 1 μg ml^−1^ LPS for 24 h. The expression of miR-200c-3p was detected by qRT-PCR. Native, THP1 cells were treated with the solvent used. (**h**) THP1 cells were treated with CAPE or JSH-23 and then treated with 1 μg ml^−1^ LPS for 24 h. The expression of miR-200c-3p was detected by qRT-PCR. Native, THP1 cells were treated with the solvent used. (**i**) THP1 cells were transfected with the corresponding siRNAs and then treated with 10 μg ml^−1^ LTA for 24 h. The expression of miR-200c-3p was detected by qRT-PCR. Native, THP1 cells were treated with the solvent used. (**j**) THP1 cells were treated with CAPE or JSH-23 and then treated with 10 μg ml^−1^ LTA for 24 h. The expression of miR-200c-3p was detected by qRT-PCR. Native, THP1 cells were treated with the solvent used. The data are shown as the mean±s.e.m. *P<0.05 and **P<0.01.

The transcription factor NF-κB heterodimer (p65/RelA and p50) has an important role in cellular responses to viral and bacterial infections [41, 42]. Besides, the determinant role of NF-κB in the regulation of miRNA expression has been revealed by previous studies [43–45]. It is well known that the activation of NF-κB signaling is essential in H5N1 infection and propagation [46–48]. Disabling the function of NF-κB heterodimer with small interfering RNAs (siRNAs) (si-p65 and si-p50) or its specific inhibitors, caffeic acid phenethyl ester (CAPE) and JSH-23, we found that the induction of miR-200c-3p by H5N1 infection was significantly repressed (Figure 4a and b and Supplementary Figure S6a). Considering that NS1 was reported to inhibit NF-κB activation, we proposed that there is an NF-κB-dependent manner for influenza virus to induce the expression of miR-200c-3p besides NS1-mediated induction of miR-200c-3p [49]. As influenza A virus infection-generated single-stranded viral RNA (vRNA) can be recognized by RIG-I to activate NF-κB and induce multiple gene expressions [43], we transfected RNA extracted from H5N1-infected cells (H5N1-vRNA) into A549 cells with Lipofectamine 2000 to test whether vRNA can induce the upregulation of miR-200c-3p. Interestingly, we found that transfection of H5N1-vRNA significantly elevated the expression level of miR-200c-3p compared with transfection of RNA extracted from AF-treated cells (Supplementary Figure S6b). In addition, repressing the function of NF-κB heterodimer with siRNAs (si-p65 or si-p50) or its specific inhibitors (CAPE and JSH-23) also resulted in the inhibition of the upregulation of miR-200c-3p induced by H5N1-vRNA transfection (Figure 4c and d and Supplementary Figure S6c), suggesting that H5N1-vRNA-induced expression of miR-200c-3p requires NF-κB activation.

Previous studies have established that NF-κB signaling activation is crucial in cellular responses to stimuli such as poly (I:C), LPS and LTA [50–52]. To assess whether the induction of miR-200c-3p by poly (I:C), LPS and LTA is also mediated by NF-κB signaling pathway, we again disabled the function of NF-κB heterodimer by using siRNAs or its specific inhibitors. Knockdown of p65 and p50 significantly repressed the upregulation of miR-200c-3p induced by the treatment of poly (I:C), LPS or LTA (Figure 4e,g and i and Supplementary Figure S6d, e and f). In addition, pretreatment with CAPE or JSH-23 also blocked the induction of miR-200c-3p in cells treated with poly (I:C), LPS or LTA (Figure 4f,h and j). The knockdown efficiency of siRNAs on p65 and p50 gene expression was validated by qRT-PCR (Supplementary Figure S6g). These data suggest that the induction of miR-200c-3p by avian influenza A virus H5N1, double-stranded RNA poly (I:C), bacterial LPS and LTA is NF-κB-dependent.

### Inhibition of miR-200c-3p protects against H5N1 virus infection

**Figure 5:**
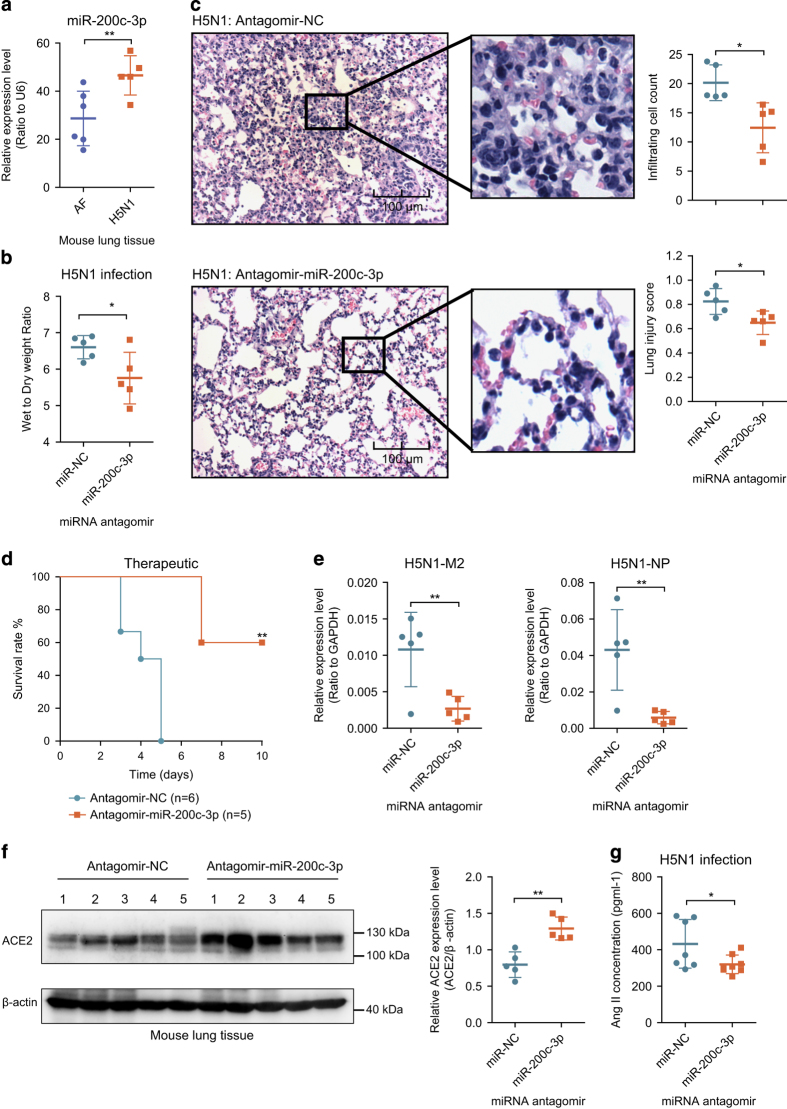
Inhibition of miR-200c-3p protects mice from H5N1 virus infection. (**a**) qRT-PCR analysis of miR-200c-3p expression in the lungs of mice challenged with AF or H5N1 virus for 3 days (1×10^6^ TCID_50_ (50% tissue culture infectious dose)). (**b**) C57BL/6 mice (*n*=5, each group) were intraperitoneally injected with antagomir of miR-200c-3p or NC (10 mg kg^−1^) 24 h before, as well as 6 and 24 h after H5N1 virus instillation (1×10^6^ TCID_50_). Wet-to-dry lung tissue weight ratios of the mice were detected 3 days after H5N1 instillation. (**c**) Representative images of lung pathology (hematoxylin and eosin) of H5N1-infected mice treated with antagomir of miR-200c-3p or NC. The number of infiltrating neutrophils per microscopic field and lung injury scores are shown in the graphs. *n*=100 fields were analyzed for five mice for each treatment. (**d**) Kaplan–Meier survival curves of C57BL/6 mice intraperitoneally injected with antagomir of miR-200c-3p or NC three times (1, 24 and 48 h, 20 mg kg^−1^) after H5N1 virus instillation (1×10^6^ TCID_50_). **P<0.01 when comparing the antagomir-NC+H5N1 group with the antagomir-miR-200c-3p+H5N1 group (log-rank test). (**e**) qRT-PCR analysis of M2 and NP mRNA relative expression levels in the lungs of mice. Lung tissues were obtained on day 3 after virus instillation. (**f**) ACE2 protein expression in the lungs of mice was detected by western blotting. Lung tissues were obtained on day 3 after virus instillation. (**g**) Ang II levels in the plasma of mice were determined using radioimmunoassays. The plasma was obtained on day 3 after virus instillation. The data are shown in the graph as the mean±s.e.m. *P<0.05 and **P<0.01.

To inhibit the function of miR-200c-3p in H5N1 virus infection, inhibitors of miR-200c-3p or an NC were transfected into A549 cells before virus infection. Inhibition of miR-200c-3p significantly blocked the downregulation of ACE2 protein induced by H5N1 virus infection (Supplementary Figure S7a). ARDS induced by H5N1 virus in mice was used to mimic and investigate the pathogenesis of H5N1-induced human ARDS [53]. H5N1 virus infection results in lung edema, inflammatory cellular infiltration and lung injury in mice [53]. Our results showed that the expression of miR-200c-3p in the lung tissue of H5N1-infected mice was also markedly upregulated (Figure 5a). Previously, we reported that the expression of ACE2 protein was downregulated in H5N1-infected mouse lung tissue [16]. As inhibition of miR-200c-3p resulted in recovery of ACE2 protein level in H5N1-infected A549 cells, miR-200c-3p may be a therapeutic target for H5N1-infected mice. In fact, administration of miR-200c-3p antagomir (24 h before, 6 and 24 h after infection, intraperitoneally 10 mg kg^−1^) indeed improved wet-to-dry lung tissue weight ratio, which indicates the degree of lung edema (Figure 5b). In mice administered with miR-200c-3p antagomir, lung injury scores (including alveolar wall thickening, hyaline membranes formatting and airspaces filling with proteinaceous debris) [54] were decreased, and inflammatory cell infiltration was alleviated (Figure 5c). Moreover, administration of miR-200c-3p antagomir (1, 24 and 48 h after infection, intraperitoneally 20 mg kg^−1^) markedly improved the survival rate of mice infected with H5N1 virus (Figure 5d) and restrained the viral replication in the lungs (Figure 5e and Supplementary Figure S7b). The body weight loss of surviving H5N1-infected mice administered with miR-200c-3p antagomir was recovered (Supplementary Figure S7c). Administration of miR-200c-3p antagomir also resulted in the recovery of ACE2 protein level in the lung tissue (Figure 5f). Accordingly, treatment with miR-200c-3p antagomir markedly reduced the plasma level of Ang II of H5N1-infected mice as well (Figure 5g). In summary, our data demonstrated that inhibiting the function of miR-200c-3p improves lung injury and ARDS induced by H5N1 virus infection, rendering miR-200c-3p as a potential therapeutic target.

## Discussion

Although influenza infection and severe pneumonia are known to cause much of their mortality by inducing ARDS, the mechanisms are still not fully understood, and effective drugs are still lacking [36, 55, 56]. Our study indicates that miR-200c-3p has a crucial role in the regulation of ACE2 in ALI or ARDS induced by H5N1 virus infection and severe pneumonia. Our previous studies have demonstrated that ACE2 protects mice against ALI induced by severe acute respiratory syndrome-coronavirus spikes, acid aspiration, sepsis and avian influenza virus [16, 18]. In this study, we showed that avian influenza virus, poly (I:C), bacterial LPS and LTA use a similar approach to reduce ACE2 level: the elevated miR-200c-3p directly downregulates ACE2 expression. Moreover, plasma levels of miR-200c-3p and its downstream effector Ang II were significantly elevated in severe pneumonia patients.

**Figure 6:**
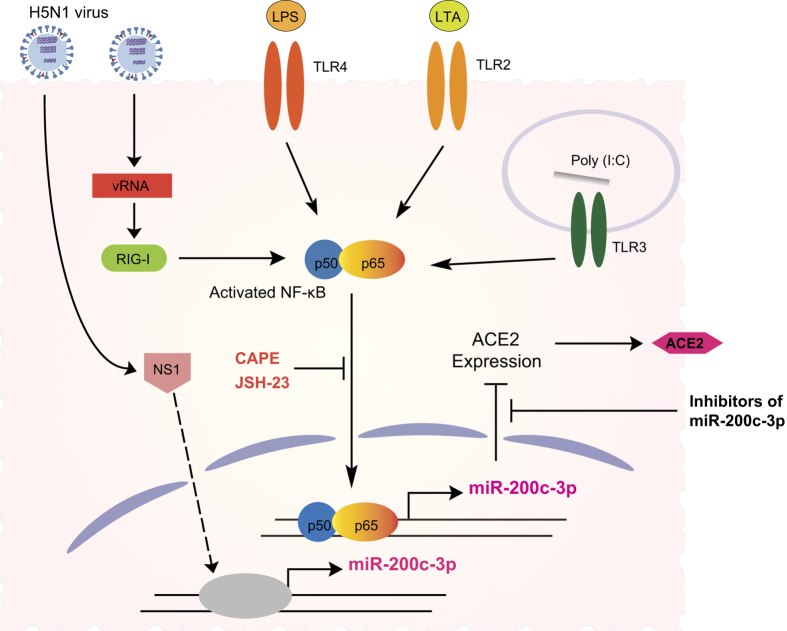
A proposed model for the regulation of miR-200c-3p in cells. H5N1 avian influenza virus uses two different ways to induce miR-200c-3p expression: one is through NS1 protein, and the other is through vRNA. H5N1-vRNA-, poly (I:C)-, bacterial LPS- and LTA-induced upregulation of miR-200c-3p requires NF-κB activation. Increased expression of miR-200c-3p directly reduces ACE2 protein expression.

While NS1 was reported to inhibit the activation of NF-κB, it is a matter of common observation that NF-κB signaling pathway is still significantly activated upon H5N1 virus infection and multiple NF-κB-dependent genes are expressed [46–49]. Interestingly, here we found that pathogenic avian influenza virus can induce the upregulation of miR-200c-3p through both the expression of NS1 protein and the accumulation of vRNA (Figure 6). NS1 is a multifunctional protein that interacts with more than 50 proteins to regulate innate immune pathways, splicing and mRNA export [57, 58]. Unlike NS1 of human pandemic H1N1/2009, NS1 of avian highly pathogenic H5N1 effectively inhibits the polyadenylation of cellular pre-mRNA in A549 cells [59]. Batista *et al*. [60] have shown that the transcription of miR-200c/141 can be induced by the bypass of the usual PTPN6 polyadenylation. NS1 of pathogenic avian influenza viruses may induce the expression of miR-200c-3p and miR-141-3p by bypassing the usual PTPN6 polyadenylation, which needed to be further investigated. Moreover, a study has reported that NA protein of influenza A/Puerto Rico/8/1934 (H1N1, PR8) can downregulate ACE2 [61]; therefore, whether NA of H5N1 and H7N9 viruses can downregulate ACE2 requires further investigation.

NF-κB is a critical regulator of defensive response to diverse pathogens. Viruses, bacteria, parasites and injury initiate distinct signal-transduction pathways to activate NF-κB. Influenza virus can induce the activation of NF-κB by the accumulation of viral products, including viral protein and vRNA [62, 63]. In this study, we found that transfection of RNA extracted from H5N1 virus-infected cells elicited the upregulation of miR-200c-3p, which can be suppressed by the loss of p65 function (Figure 6). However, the detailed molecular mechanism needs to be further investigated. The expression levels of miR-200c-3p have been found to be markedly upregulated in the livers of hepatocellular carcinoma patients [64]. Among these patients, nine patients were diagnosed with HBV infection and five patients were diagnosed with HCV infection, thus suggesting that HBV and HCV infection may induce the expression of miR-200c-3p. Whether HCV or HBV RNA can induce the upregulation of miR-200c-3p requires further study.

As we know, poly (I:C), LPS and LTA can induce the activation of NF-κB via Toll-like receptor 3, Toll-like receptor 4 and Toll-like receptor 2, respectively [65–67]. In fact, we also noticed that poly (I:C), bacterial LPS and LTA can induce the upregulation of miR-200c-3p to downregulate ACE2 protein expression in an NF-κB signaling pathway-dependent manner (Figure 6). Previous study has shown that overexpression of ACE2 protects against LPS-induced lung injury in rat [23]. The downregulation of ACE2 might be mediated by miR-200c-3p and inhibition of miR-200c-3p might protect against LPS-induced lung injury as well. The observations represented in this study may provide an example how pathogenic avian influenza viruses and bacteria use NF-κB activity.

Compared with healthy controls, the plasma levels of both miR-200c-3p and its downstream effector Ang II were much higher in severe pneumonia patients. As previous studies have reported that angiotensin-converting enzyme inhibitors, which reduce the production of Ang II, and Ang II receptor blockers, which block the function of Ang II, do have some beneficial effects on pneumonia-related clinical outcomes [68, 69], inhibition of miR-200c-3p may also produce a positive clinical outcome for pneumonia. In addition, previous study has reported that the miR-200c-3p level was positively related to the severity of interstitial lung disease [70]. Further studies are needed to explore whether plasma levels of miR-200c-3p or Ang II can serve as a biomarker or therapeutic target for severe pneumonia.

In recent years, miRNA-based strategies for exploring novel therapeutic drugs have experienced rapid development. Several miRNA-targeting drugs have now entered clinical testing or are even close to gaining access to markets [28]. We found that administration of antagomirs of miR-200c-3p can ameliorate ALI and lung edema in mice induced by H5N1 virus infection, suggesting a potential miRNA-targeting drug for avian influenza viruses-mediated ALI. One study has demonstrated that administration of anti-miR-200c-5p oligonucleotide attenuates pulmonary inflammatory responses and lung injury by inhibiting the downregulation of dual-specificity phosphatase 1 in an LPS-induced mouse model [71]. In accordance with our result, ACE2 was not a target of miR-200c-5p (Figure 2b).

Collectively, we found that NS1 and vRNA of avian influenza virus can induce the upregulation of miR-200c-3p to downregulate ACE2 protein expression. NF-κB-dependent induction of miR-200c-3p is a shared mechanism of H5N1-vRNA, poly (I:C), LPS and LTA induced ACE2 reduction, suggesting a molecular mechanism of ALI/ARDS caused by bacterial and viral infections: the elevated miR-200c-3p downregulated ACE2 expression, increased Ang II levels and caused lung injury through Ang II type 1 receptor. Consistently, blocking the function of miR-200c-3p protects H5N1-infected mice from ALI, indicating miR-200c-3p as a potential therapeutic target.

## Materials and Methods

### Influenza viruses

The influenza viruses A/Jilin/9/2004 (H5N1) and A/New Caledonia/20/1999 (H1N1) were used in this study. All live influenza virus experiments were performed in biosafety level 3 facilities according to governmental guidelines.

### Cell culture

A549 cells (human lung adenocarcinoma epithelial cells) were purchased from the American Type Culture Collection (ATCC, Rockville, MD, USA) and cultured in Ham's F12 nutrient medium (HyClone, Logan, UT, USA). HEK293T and THP1 cells were purchased from the Peking Union Medical College Cell Culture Center (Beijing, China). HEK293T cells were propagated in Dulbecco’s modified Eagle’s medium (HyClone). THP1 cells were cultured in RPMI-1640 medium (HyClone). Cells were cultured in the appropriate medium supplemented with 10% (v v−1) fetal bovine serum (Gibco, Grand Island, NY, USA), 100 U ml^−1^ penicillin and 100 U ml^−1^ streptomycin at 37 °C.

### miRNA-seq data analysis

Infected with H1N1 or H5N1 virus (MOI=4) or AF control for 18 h, A549 cells were collected using TRIzol Reagent (Invitrogen, Carlsbad, CA, USA). Total RNA of the cells was isolated following the standard protocol. RNA libraries were sequenced using an Illumina HiSeq 2500 Platform (Illumina, San Diego, CA, USA) with single-end (SE) 50 nt (SE50), and each sample yielded an average of 20 M reads. The software fastx_toolkit (version: fastx_toolkit_0_0_13) was used to preprocess small RNA-seq raw data. The software Bowtie2 (version: Bowtie2-2.1.0) and Rfam database (http://rfam.xfam.org/) were used to filter ribosomal RNA or transfer RNA. The commands mapper.pl and miRDeep2.pl in the software miRDeep2 (version: miRDeep2_0_0_7) were used for mapping sample reads to the human genome (version hg19, http://hgdownload.cse.ucsc.edu/downloads.html) and detecting conserved miRNA.

### Quantitative real-time PCR

Following the standard protocol, total RNA was isolated in RNase-free environment using TRIzol Reagent (Invitrogen). For gene expression analysis, cDNA was synthesized from 1.5 μg of total RNA with random primers using the High-Capacity cDNA Reverse Transcription Kit (Applied Biosystems, Foster City, CA, USA). For miRNA expression analysis, cDNA was synthesized with stem-looped miRNA-specific reverse transcription primers by using the same kit. Amplified products were detected using the FastStart Universal SYBR Green Master Mix II (Roche, Basel, Switzerland) on a LightCycler 480 PCR System (Roche). The relative gene expression levels were normalized to the reference gene, human glyceraldehyde-3-phosphate dehydrogenase (*GAPDH*). The relative miRNA expression levels were normalized to small non-coding RNA U6. The specific primers used were synthesized by Invitrogen and listed in Supplementary Table S2.

### Cell viability assays

The A549 cells were infected with H1N1 or H5N1 influenza virus titrated to an MOI of 4 or an equal volume of vehicle. Cell viability was determined by an MTT assay (Promega, Madison, WI, USA) at 1, 3, 6, 9, 12, 18, 24 and 36 h postinfection in the A549 cell line.

### Western blot analysis

A549 cells were transfected with 50 nM mimics of NC (5′- UUGUACUACACAAAAGUACUG-3′), miR-200c-3p (5′- UAAUACUGCCGGGUAAUGAUGGA-3′) and miR-141-3p (5′- UAACACUGUCUGGUAAAGAUGG-3′), or inhibitors of NC (5′- CAGUACUUUUGUGUAGUACAA-3′), miR-200c-3p (5′- UCCAUCAUUACCCGGCAGUAUUA-3′) and miR-141-3p (5′- CCAUCUUUACCAGACAGUGUUA-3′) for 36 h. The mimics and inhibitors were synthesized by GenePharma (Shanghai, China). Then, the cells were collected with RIPA lysis buffer. Protein was detected by western blotting following the procedures described previously [72]. Anti-ACE2 antibody was purchased from Abcam (Cambridge, MA, USA; ab108252). Antibodies against β-actin (clone AC-15, A5441) and Flag tag (F7425) were purchased from Sigma-Aldrich, St Louis, MO, USA.

### Luciferase activity assay

The wild-type and mutant-type 3′-UTRs of the ACE2 transcript were cloned into the psiCHECK-2 vector (Promega) through the *Not*I and *Xho*I restriction enzyme cutting sites. miRNA mimics were transfected into HEK293T cells using Lipofectamine RNAiMAX (Invitrogen). After 24 h, 1 μg of wild-type vector or mutant vector was transfected into HEK293T cells using Lipofectamine 2000 (Invitrogen). At 48 h after transfection of miRNA mimics, relative luciferase activity was calculated according to the manufacturer’s instructions. Wild-type or mutant-type miR-200c-3p mimics were transfected into HEK293T cells using Lipofectamine RNAiMAX (Invitrogen). After 24 h, 1 μg of wild-type vector was transfected into HEK293T cells using Lipofectamine 2000 (Invitrogen). At 48 h after transfection of miRNA mimics, relative luciferase activity was calculated according to the manufacturer’s instructions.

### Plasmids

H5N1 influenza viral protein coding genes, hemagglutinin, NA, NS1, nonstructural protein 2, polymerase complex PA, polymerase complex PB1, polymerase complex PB2, M1, M2 and NP were cloned into the Peak13 vector (provided by B Seed, Harvard Medical School, Boston, MA, USA). Coding genes of NS1 protein of H1N1 and H7N9 influenza virus were also cloned into the Peak13 vector.

### vRNA, poly (I:C), LPS and LTA experiments

Poly (I:C) (P0913; Sigma, St Louis, MO, USA) was transfected into A549 cells with Lipofectamine 2000 for 6 h. LPS (from *Escherichia coli* O111:B4, L4391; Sigma) or LTA (from *Staphylococcus aureus*, L2515; Sigma) were added to A549 and THP1 cells for 24 h. RNA extracted from A549 cells challenged with H1N1 virus, H5N1 virus or AF control was transfected into A549 cells with Lipofectamine 2000 for 12 h. Then, RNA and protein samples were collected and analyzed.

### Clinical specimens

Severe pneumonia patients were recruited at the Peking Union Medical College Hospital (PUMCH, Beijing, China). Healthy volunteers were recruited as controls. All participants had written informed consent. With the approval of the Institutional Review Board of PUMCH, this study was performed following the ethical guidelines of the 1975 Declaration of Helsinki. The concentration of Ang II in plasma was detected using the ELISA Kit (RapidBio Lab, Calabasas, CA, USA).

### Plasma miRNA extraction and detection

miRNAs were extracted from 400 μl plasma using an miRNeasy Serum/Plasma Kit (Qiagen, Hilden, Germany). A synthetic spike-in control, *Caenorhabditis elegans miR-39* (cel-miR-39), was added to the lysed samples for internal normalization. cDNA was synthesized using the TaqMan MicroRNA Assays Kit and High-Capacity cDNA Reverse Transcription Kit (Applied Biosystems). PCR reactions were performed on a LightCycler 480 PCR system (Roche) using the TaqMan Universal Master Mix II (Applied Biosystems).

### RNA interference knockdown

NF-κB p65/RelA-specific siRNA, p50-specific siRNA and nonspecific control siRNA were synthesized by GenePharma. The p65 siRNA duplexes (siRNA1: 5′- GGACAUAUGAGACCUUCAA-3′; siRNA2: 5′- CUUCCAAGUUCCUAUAGAA-3′), the p50 siRNA (5′- CGCCAUCUAUGACAGUAAAUU-3′) or a control siRNA were transfected into cells (with a final concentration as 100 μM) with Lipofectamine RNAiMAX (Invitrogen) for 24 h, and then subsequent experiments were performed.

### Inhibition of NF-κB transcriptional activity

CAPE (S7414) and JSH-23 (S7351) were purchased from Selleck Chemicals (Houston, TX, USA) and resolved in dimethyl sulfoxide. CAPE and JSH-23 were added to cells (with a final concentration of 75 and 100 μM, respectively) for 2 h, and then subsequent experiments were performed.

### *In vivo* experiments

Wild-type C57BL/6 mice were purchased from Vital River (Beijing, China). Antagomirs of NC (5′- CAGUACUUUUGUGUAGUACAA-3′) and miR-200c-3p (5′- UCCAUCAUUACCCGGCAGUAUUA-3′) (10 mg kg^−1^; GenePharma) were intraperitoneally injected into C57BL/6 mice 24 h before, and 6 and 24 h after intratracheal instillation of H5N1 virus. At 3 days postinfection, the mice were killed, and lung injury was assessed as described previously [16]. For the assessment of survival rates, virus replication and Ang II, antagomirs of NC and miR-200c-3p (5′- UCCAUCAUUACCCGGCAGUAUUA-3′) (20 mg kg^−1;^ RiboBio, Guangzhou, China) were intraperitoneally injected into C57BL/6 mice 1, 24 and 48 h after intratracheal instillation of H5N1 virus. The animal experiments were performed in the Institute of Military Veterinary Medicine, Academy of Military Medical Sciences following governmental and institutional guidelines.

### Statistical analysis

Unpaired *t*-tests were used for the cell culture and luciferase reporter assay data analysis. Pearson's correlation analysis was used to analyze the relationship between miRNAs and virus-infected cell viability or viral replication. The Mann–Whitney *U*-test was used for the analysis of clinical data. All statistical tests were calculated using GraphPad Prism 5.0 (La Jolla, CA, USA). A two-tailed *P*-value <0.05 was considered statistically significant.
