# Supplementary material for: miRNA-200c-3p is crucial in acute respiratory distress syndrome
Source: Cell Discov. 2017 Jun 27;3:17021–. doi: 10.1038/celldisc.2017.21 (PMC5485385; doi:10.1038/celldisc.2017.21)
Supplement: Supplementary Figures [file celldisc201721-s1.pdf]

Supplemental Figure S1

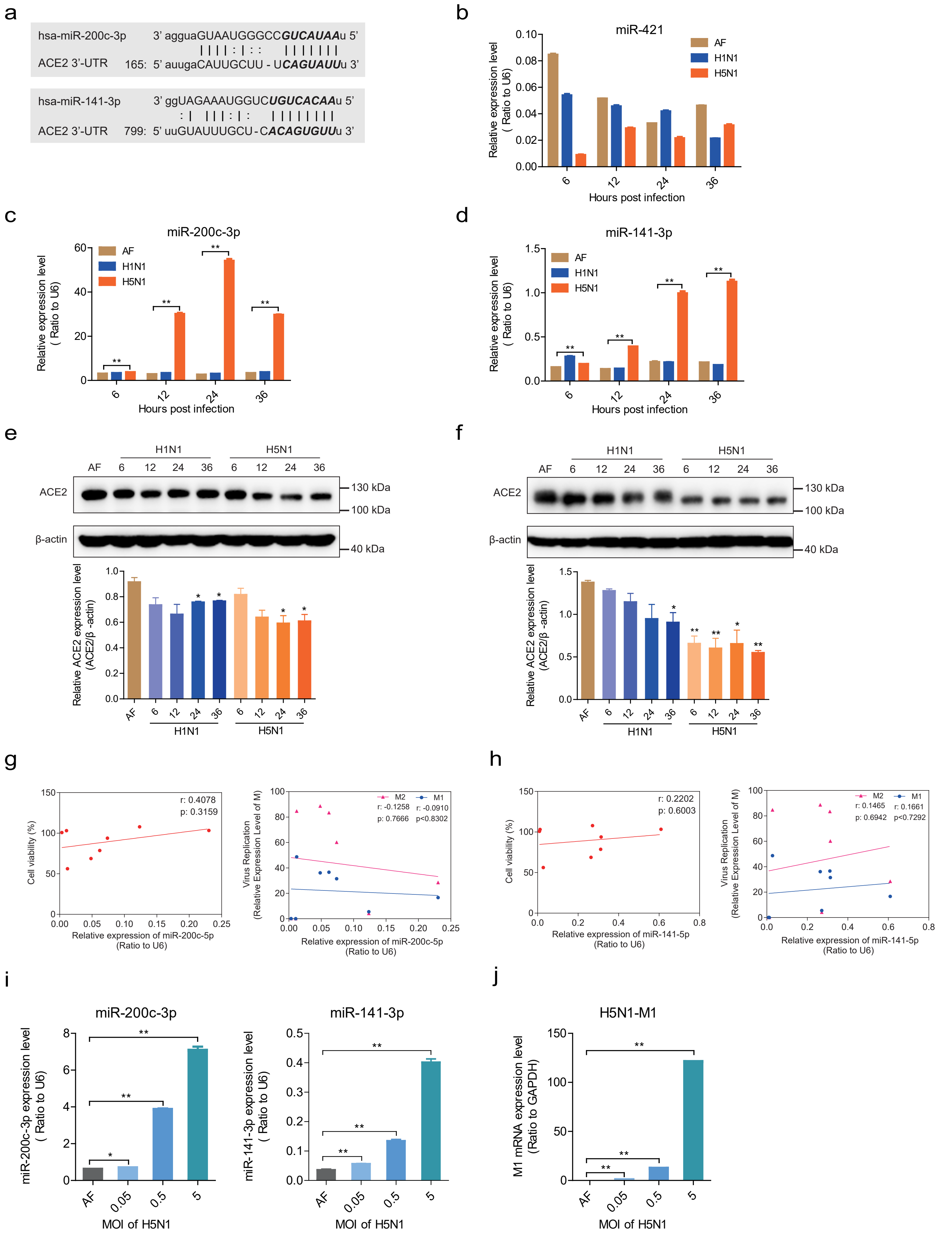

Supplemental Figure S2

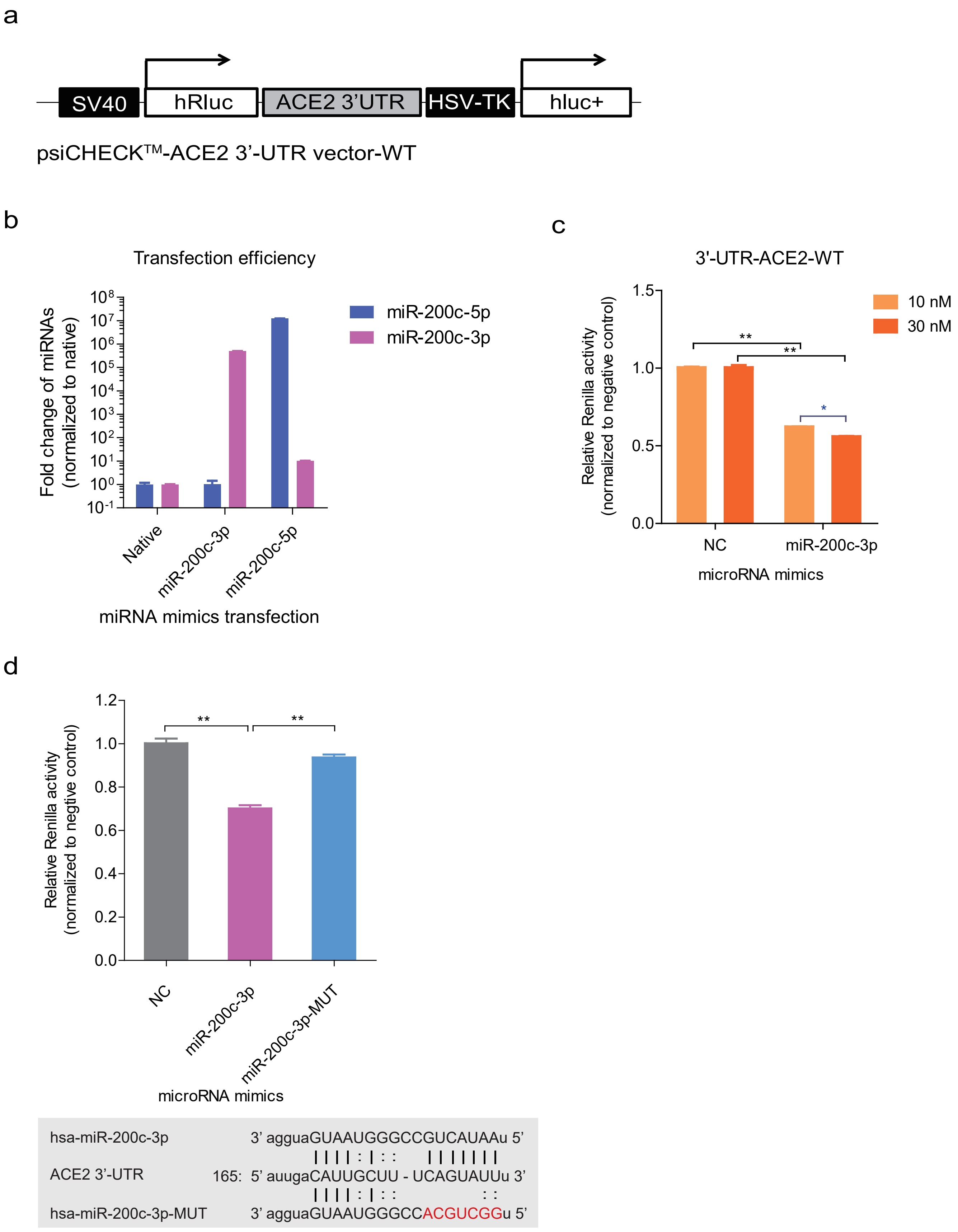

Supplemental Figure S3

a

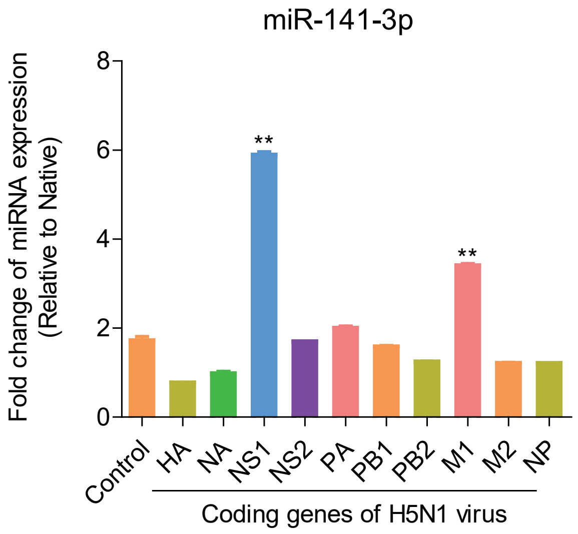

b

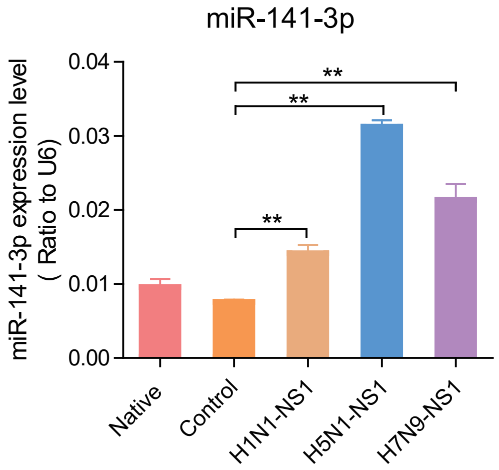

c

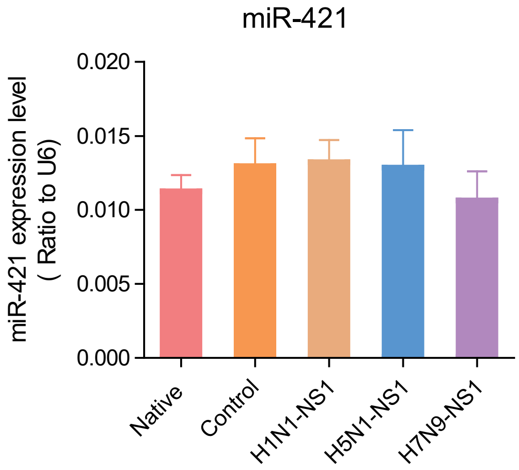

Supplemental Figure S4

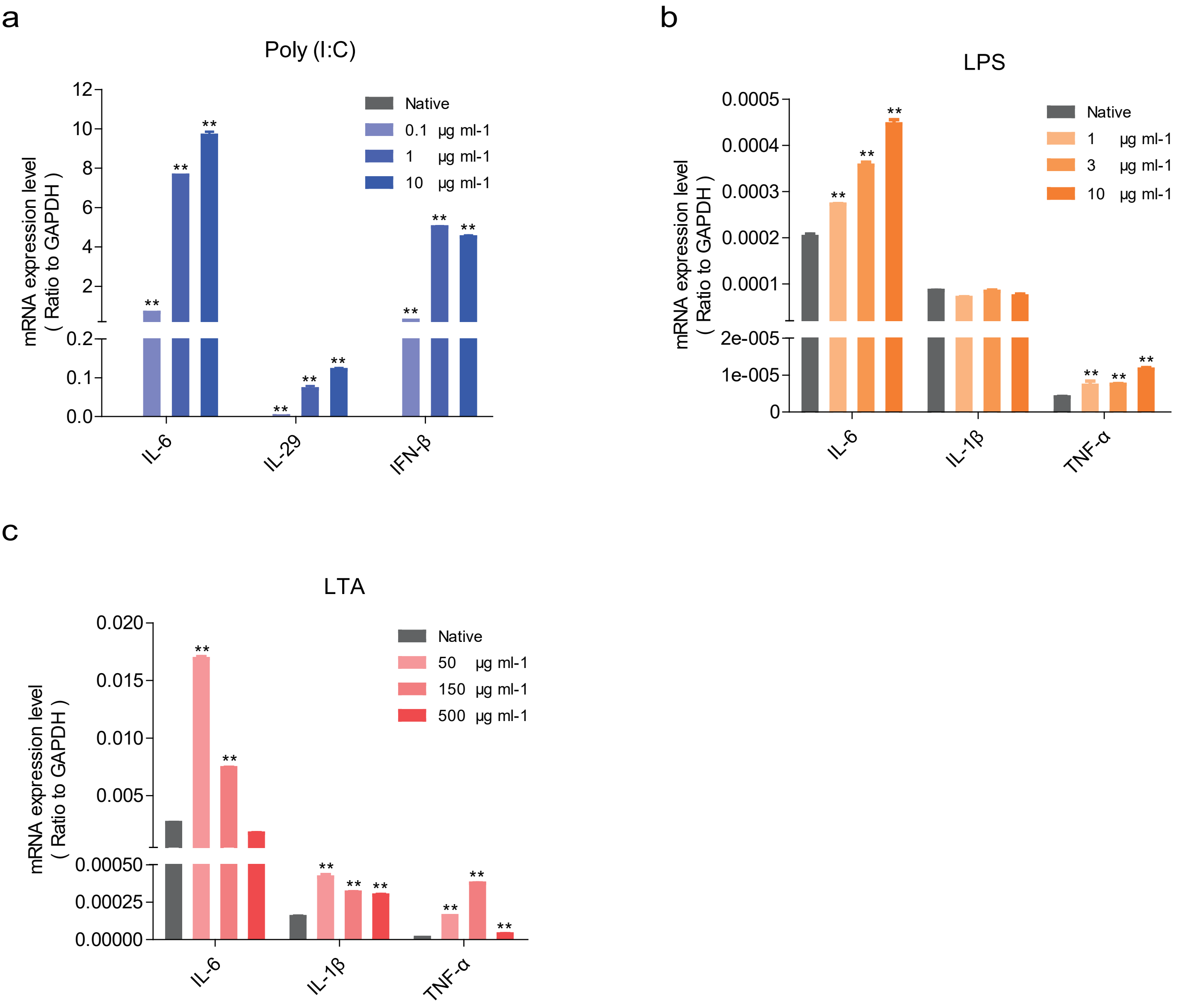

Supplemental Figure S5

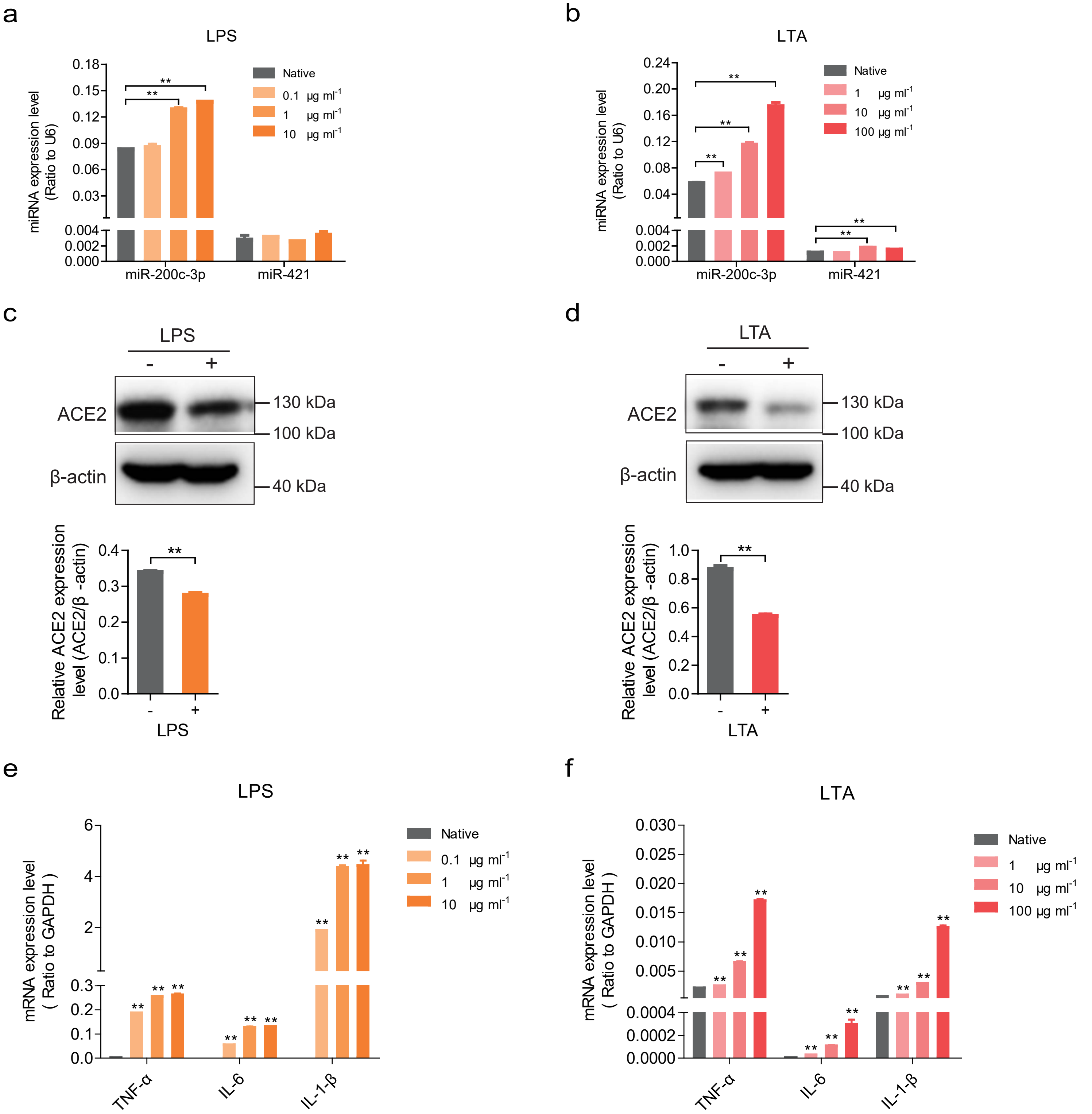

Supplemental Figure S6

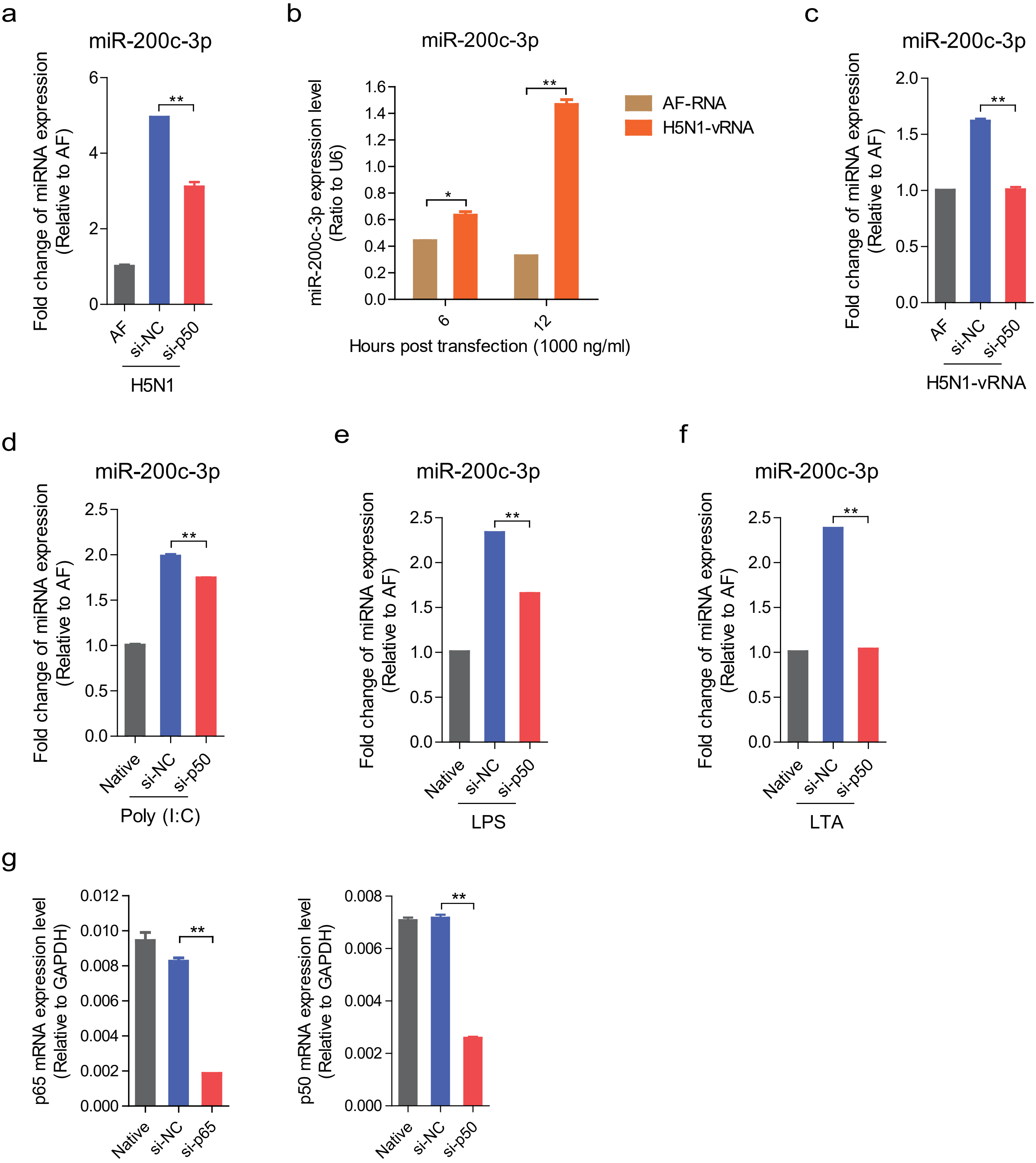

Supplemental Figure S7

a

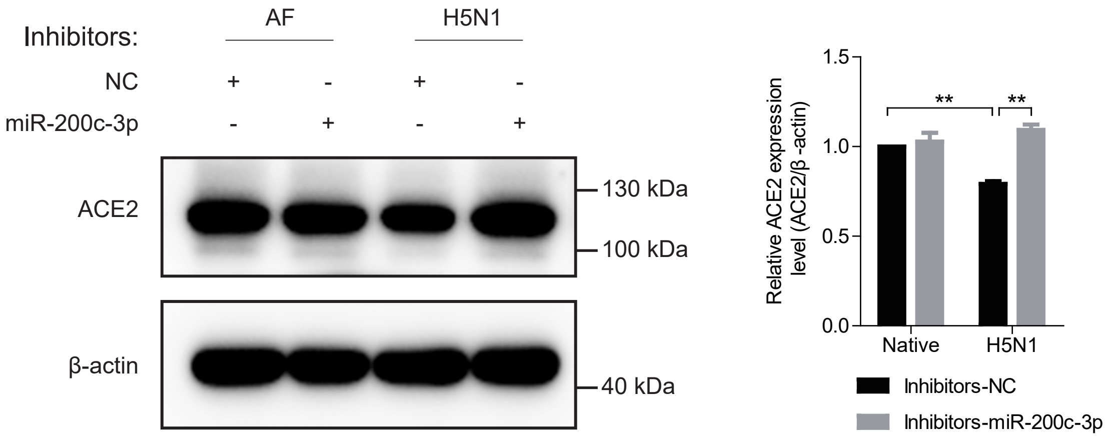

b

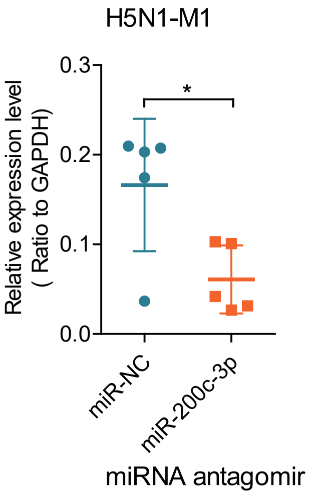

c

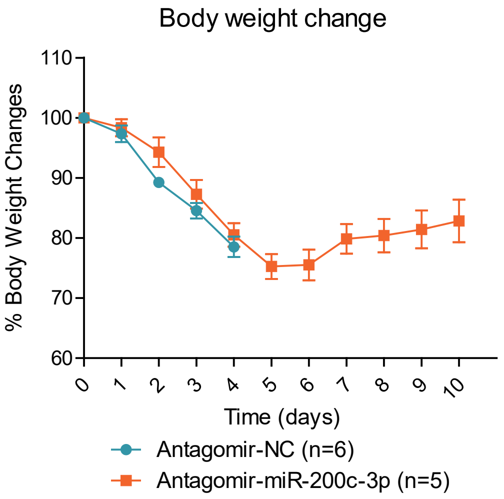

Supplementary Figure Legends

Supplementary Figure S1. Expression of miRNAs and ACE2 in A549 cells and HEK293T cells infected with H1N1 or H5N1 virus. (**a**) The predicted binding sites on the 3'-UTR of ACE2 for miR-200c-3p and miR-141-3p. (**b**) qRT-PCR analysis of miR-421 in A549 cells after challenged with H1N1 virus, H5N1 virus (MOI=4) or AF control at the indicated hours. (**c, d**) qRT-PCR analysis of the expression of miR-200c-3p and miR-141-3p in HEK293T cells after challenged with H1N1 virus, H5N1 virus (MOI=4) or AF control at the indicated hours. (**e, f**) Western blotting analysis of ACE2 protein expression in A549 cells and HEK293T cells after challenged with H1N1 virus, H5N1 virus (MOI=4) or AF control at the indicated hours. β-actin served as an internal control. **(g)** Correlation between the expression of miR-200c-5p and cell viability or virus replication in H5N1-infected A549 cells. Pearson correlation analysis was used to analyze the correlation. Pearson correlation coefficients (r) and P-values are provided in each graph. Viral replication was indicated with the relative expression levels of matrix protein 1 (M1) and matrix protein 2 (M2). (**h**) Correlation between the expression of miR-141-5p and cell viability or virus replication in H5N1-infected A549 cells. Pearson correlation analysis was used to analyze the correlation. Pearson correlation coefficients (r) and P-values are provided in each graph. (**i**) qRT-PCR analysis of the expression of miR-200c-3p and miR-141-3p in A549 cells infected with H5N1 virus for 48 h at the indicated multiplicity of infection (MOI). (**j**) qRT-PCR analysis of the expression of M1 in A549 cells infected with H5N1 virus for 48 h at the indicated MOI. The data are shown as the mean±SEM. *P<0.05, **P<0.01.

Supplementary Figure S2. miR-200c-3p targets 3'-UTR of ACE2. (**a**) A reporter vector was constructed in which the ACE2 3'-UTR was fused downstream of the *Renilla* luciferase coding gene under the control of the SV40 promoter (ACE2 3'-UTR-WT). (**b**) Transfection efficiency of miR-200c-5p mimics and miR-200c-3p mimics was detected by qRT-PCR. Native, untreated HEK293T cells. (**c**) Various amounts of miR-200c-3p were co-transfected with the reporter vector into HEK293T cells. Forty-eight hours later, luciferase activity was measured by the Dual Luciferase Reporter Assay system. (**d**) Mimics of negative control (NC), miR-200c-3p and mutant miR-200c-3p (miR-200c-3p-mut) were transfected into HEK293T cells respectively. Then ACE2 3'-UTR-WT reporter vector was transfected into the cells. Luciferase activity was detected using the Dual Luciferase Reporter Assay system. The data are shown as the mean±SEM. $^*P<0.05$, $^{**}P<0.01$.

Supplementary Figure S3. Expression of miR-141-3p and miR-421 in HEK293T cells overexpressing coding genes of influenza virus. (**a**) Vectors containing H5N1 influenza viral protein coding genes (HA, NA, NS1, NS2, PA, PB1, PB2, M1, M2, and NP) were individually transfected into HEK293T cells for 48 h. The expression of miR-141-3p was quantified by qRT-PCR. The empty vector served as a control. Native, untreated HEK293T cells. (**b, c**) Vectors coding for NS1 of H1N1, H5N1 and H7N9 viruses were transfected into HEK293T cells for 72 h. The expression of miR-141-3p and miR-421 was detected by qRT-PCR. The data are shown as the mean±SEM. $^{**}P<0.01$.

Supplementary Figure S4. The induction of inflammatory cytokines in poly (I:C), LPS

and LTA-treated A549 cells. (**a**) qRT-PCR analysis of mRNA expression of IL-6, IL-29 and IFN-β in A549 cells treated with poly (I:C) for 6 h at the indicated concentration. (**b**) qRT-PCR analysis of mRNA expression of IL-6, IL-1β and TNF-α in A549 cells treated with LPS for 24 h at the indicated concentration. (**c**) qRT-PCR analysis of mRNA expression of IL-6, IL-1β and TNF-α in A549 cells treated with LTA for 24 h at the indicated concentration. The data are shown as the mean±SEM. **P<0.01.

Supplementary Figure S5. LPS and LTA induce the expression of miR-200c-3p in THP1 cells. (**a, b**) qRT-PCR analysis of the expression of miR-200c-3p and miR-421 in THP1 cells treated with LPS or LTA for 24 h. (**c, d**) ACE2 protein expression in THP1 cells challenged with LPS (10 μg/ml) and LTA (500 μg/ml) was also detected. β-actin served as an internal control. (**e, f**) qRT-PCR analysis of mRNA expression of TNF-α, IL-6 and IL-1β in THP1 cells challenged with LPS or LTA for 24 h at the indicated concentration. The data are shown as the mean±SEM. **P<0.01.

Supplementary Figure S6. Knockdown of p50 suppressed the induction of miR-200c-3p. (**a**) A549 cells were transfected with the corresponding siRNAs and then challenged with AF or H5N1 (MOI=4) for 48 h. The expression of miR-200c-3p was detected by qRT-PCR. (**b**) The expression of miR-200c-3p in A549 cells transfected with 1000 ng/ml RNA extracted from AF- or H5N1-challenged cells for 12 h was detected by qRT-PCR. (**c**) A549 cells were transfected with the corresponding siRNAs and then transfected with 1 μg/ml RNA extracted from AF- or H5N1-infected cells for 12 h. The expression of miR-200c-3p was detected by

qRT-PCR. (**d**) A549 cells were transfected with the corresponding siRNAs and then transfected with 1 μg/ml poly (I:C) for 12 h. The expression of miR-200c-3p was detected by qRT-PCR. (**e**) THP1 cells were transfected with the corresponding siRNAs and then treated with 1 μg/ml LPS for 24 h. The expression of miR-200c-3p was detected by qRT-PCR. (**f**) THP1 cells were transfected with the corresponding siRNAs and then treated with 10 μg/ml LTA for 24 h. The expression of miR-200c-3p was detected by qRT-PCR. (**g**) The expression of p65 or p50 mRNA in A549 cells transfected with 100 μM control siRNA and p65- or p50-specific siRNA. The data are presented as mean±SEM. **P<0.01.

Supplementary Figure S7. Inhibition of miR-200c-3p ameliorates H5N1 virus infection. (**a**) After transfected with inhibitors of negative control (NC) or miR-200c-3p for 6 h, A549 cells were infected with H5N1 virus (MOI=4) or mock-infected with AF. Twenty-four hours post infection, the cells were collected and the expression of ACE2 protein was detected. β-actin served as an internal control. (**b**) qRT-PCR analysis of M1 in the lung of H5N1-infected mice treated with the antagomir of negative control (NC) or miR-200c-3p. (**c**) Mice were administrated with antagomir of miR-200c-3p and NC (20 mg kg^{-1}) 1 h, 24 h and 48 h after H5N1 virus instillation (1×10^6 $TCID_{50}$). Body weights were recorded for a 10 day period after H5N1 infection. The data are presented as mean±SEM. *P<0.05, **P<0.01.
